# Orodispersible Film Based on Maltodextrin: A Convenient and Suitable Method for Iron Supplementation

**DOI:** 10.3390/pharmaceutics15061575

**Published:** 2023-05-23

**Authors:** Irma Elisa Cupone, Giuliana Roselli, Fabio Marra, Marika Riva, Silvia Angeletti, Laura Dugo, Silvia Spoto, Marta Fogolari, Andrea Maria Giori

**Affiliations:** 1 Ibsa Farmaceutici Italia, Cassina de’ Pecchi, 20051 Milan, Italy; 2 Ibsa Farmaceutici Italia, 26900 Lodi, Italy; 3 Operative Research Unit of Clinical Laboratory, University of Rome Campus Bio-Medico, 00128 Rome, Italy; 4 Department of Science and Technology for Sustainable Development and One Health, University Campus Bio-Medico of Rome, 00128 Rome, Italy; 5 Department of Diagnostic and Therapeutic Medicine, University Campus Bio-Medico of Rome, 00128 Rome, Italy

**Keywords:** orodispersible film, food supplement, iron, bioavailability, maltodextrin, patient acceptability, convenient dosage form

## Abstract

Orodispersible film (ODF) is an innovative dosage form used to administer drugs and nutrients, designed to disintegrate or dissolve in the oral cavity without needing water. One of the advantages of ODF is that it is suitable for administration in older people and children who have difficulty swallowing because of psychological or physiological deficiencies. This article describes the development of an ODF based on maltodextrin, which is easy to administer, has a pleasant taste, and is suitable for iron supplementation. An ODF containing 30 mg of iron as pyrophosphate and 400 µg of folic acid (iron ODF) was developed and manufactured on an industrial scale. The kinetic profile for serum iron and folic acid upon consumption of ODF compared with a Sucrosomial^®^ iron capsule (known for its high bioavailability) was evaluated in a crossover clinical trial. The study was conducted in nine healthy women, and the serum iron profile (AUC_0–8_, T_max,_ and C_max_) of both formulations was defined. Results showed that the rate and extent of elemental iron absorption with iron ODF was comparable to that obtained using the Sucrosomial^®^ iron capsule. These data represent the first evidence of iron and folic acid absorption concerning the newly developed ODF. Iron ODF was proven to be a suitable product for oral iron supplementation.

## 1. Introduction

Iron deficiency (ID) is the most common nutritional deficiency worldwide [1]. It typically occurs because of low iron consumption, increased requirement for iron, or excessive loss of iron. Populations with the highest iron demand include children, adolescents, and women of reproductive age, particularly during pregnancy [2]. In the case of children and adolescents, the increased need for iron is related to the rapid growth of this population. Pregnant women require more iron to sustain the development of maternal and fetal tissues and to increase their hemoglobin mass in proportion to the increased need for oxygen transport [2]. For women of reproductive age, menstruation is the main cause of iron loss, which requires adequate and constant replenishment [3]. Beyond children, adolescents, and women, athletes, people on low-meat diets, and older people are particularly prone to experiencing an iron deficiency. Athletes are frequently iron deficient due to their high training schedules, resulting in increased iron loss and reduced absorption [4]. Iron deficiency is highly prevalent in people on low-meat or unbalanced diets; in vegans and vegetarians, for instance, the bioavailability of iron is usually low because of the absence of meat and fish in their diet and the high consumption of foods containing inhibitors of iron absorption, such as phytates and polyphenols. In older people, iron deficiency is often multifactorial and typically due to inadequate dietary consumption, malabsorption, occult bleeding, or the use of medications [5]. In all groups, iron deficiency adversely affects physical capacity, cognitive performance, and immunological defense. For iron deficiency correction, different strategies can be carried out including diet modification or diversification, iron fortification of foods, or iron supplementation [1]. Food supplements are gaining importance due to their capacity to support physiological functions of the body in a targeted manner, thus leading to improved well-being or reduced risk of disease. The European Directive 2002/46/EC [6] defines food supplements as “concentrated sources of nutrients (i.e., vitamins and minerals) or other substances with a nutritional or physiological effect that are marketed in dose form such as pills, tablets, capsules, liquids in measured doses.” Treatment with food supplements is often long-term and required daily. For this reason, a food supplement must be easy to take with good organoleptic properties and limited side effects to promote adherence to the treatment. Many available iron-based supplements on the market differ according to the type and quantity of iron, required frequency of administration, or dosage forms (drops, capsules, tablets, syrup, etc.). Some products contain exclusively iron whilst others associate iron with additional nutrients, such as vitamin B12, folic acid, or vitamin C. Despite its broad demand, iron supplementation is not free of drawbacks, including the unpleasant metallic taste of typical iron sources, such as ferrous fumarate and ferrous sulfate, as well as gastrointestinal discomfort. These issues are particularly challenging for children. Moreover, existing iron supplements are often in inconvenient solid forms and unsuitable for supplementation in children or adults with swallowing difficulties. Given the high prevalence of ID and since supplementation must meet the needs of a large and diverse population, including vulnerable groups, convenient and effective methods of supplementation are beneficial.

Orodispersible film (ODF) is an innovative dosage form to administer drugs and nutrients. ODFs are thin, flexible sheets to place in the oral cavity where they disintegrate or dissolve without needing water. This dosage form is especially beneficial for older people and children with difficulty swallowing for psychological or physiological deficiencies [7,8,9,10,11,12,13]. ODFs were previously demonstrated to be superior to other solid dosage forms [14] and even liquid forms such as syrup [15]. Studies demonstrated that ODF is an adequate dosage form for infants and preschool children [16,17,18] for ease of swallowing but also for flexible manufacturing that allows prompt adjustment of the size of the film. Due to these properties, ODFs have recently been proposed as an extemporaneous preparation for personalized therapy [19,20,21,22,23]. ODF is an accurate dosage form suitable for long-term daily treatment, and if packaged as a single dose, it is easy for an individual to carry. The pharmaceutical use of ODFs is now widespread while the use of ODF technology for food supplements remains limited, with only a few examples reported in the literature [24,25].

This article describes the development, scale-up, and application in the clinical setting of an ODF for iron supplementation based on maltodextrin (MDX). Iron ODF is a food supplement developed according to European regulations regarding food supplements. MDX is the polymeric substance that acts as a film-forming agent, and it is a very useful polymer for ODF development [26]. MDXs are cheap, water soluble, non-toxic polymers used extensively in pharmaceutical and food industries [27,28,29,30]. The main technical issues of ODFs are the loadable number of active substances and the unpleasant taste of most active substances [31]. The development and production of the iron ODF represented a key challenge because the intended dosage of iron to load was high (up to 30 mg/day of elemental iron) and the taste of iron is notoriously unpleasant and difficult to mask. Regarding iron supplementation, the terms ‘bioavailability’ and ‘absorption’ are often used interchangeably, even though, according to its definition, bioavailability refers to the amount of iron absorbed and incorporated into erythrocytes [32]. The bioavailability of iron preparations is typically low and reported to be less than 15%. Bioavailability depends on the type and amount of the iron salt used and the delivery system, but also on external contributing factors such as the co-administration of certain drugs (i.e., proton pump inhibitors or antacids), meals, or the presence of an inflammatory status at the gut level [33]. Therefore, we aimed to assess the absorption of elemental iron within the MDX-based ODF in a pilot crossover, open-label, single-center, comparative clinical trial. The kinetics of absorption of the two functional ingredients, elemental iron and folic acid, were evaluated in nine healthy women after a single dose of iron ODF. The Sucrosomial^®^ iron capsule was chosen as the reference product due to its high iron bioavailability, as demonstrated in clinical and pre-clinical studies [33,34]. All participants received a single dose of both supplements with a seven-day wash-out period between the two administrations, and the rate and the extent of iron and folic acid absorption were assessed.

## 2. Material and Methods

### 2.1. Film Preparation and Characterization

#### 2.1.1. Materials

Ferric pyrophosphate, ferrous lactate, ferrous bisglycinate, ferrous citrate (Dr. Paul Lohmann, Emmerthal, Germany), MDX (Roquette, Lestrem, France), glycerol, (Oleon, Ertvelde, Belgium), sorbitol (ACEF, Fiorenzuola D’Arda, Italy), lemon flavor (Kerry, Tralee, Ireland), copovidone and polysorbate 80 (Eigenmann & Veronelli, Rho, Italy), folic acid (DSM, Maastricht, The Netherlands), glyceryl monolinoleate (Gattefossè, Saint-Priest, France), sucralose (Sunvision Sweet, Xintai City, Shandong, China), citric acid and sodium citrate (Citrique Belge, Tienen, Belgium), sodium cyclamate (Faravelli, Milan, Italy), acesulfame potassium salt (Celanese, Dallas, TX, USA). All ingredients used were food grade in accordance with European regulations.

#### 2.1.2. Film Preparation

The iron ODF was manufactured by the casting method consisting of different steps. Generally, the mass was prepared under a controlled temperature and stirring speed conditions within a mixer equipped with an anchor and high-speed disk. Afterwards, the mixture was spread and dried in a drying tunnel with a controlled temperature, air circulation, and coating speed, and the slitting step then followed. In the last step, the films were punched, pouched, and sealed in suitable single-dose sachets using the pouch forming and sealing machine Speedex ACSU (R.C.A. Bignami, Varese, Italy).

#### 2.1.3. Rheological Properties of ODF Wet Mixture

To investigate the rheological properties of the mixture, the relationship between the dynamic viscosity (η) and the applied shear rate (γ̇) was evaluated using a single-step rotational test. Thixotropy was investigated using a 3-step rotational test. All tests were performed using a rheometer MCR702e (Anton Paar, Graz, Austria) equipped with a concentric cylinder (CC28.4) measuring system at a constant temperature of 25 °C. The rotational test to evaluate η was carried out by increasing γ̇ from 0.01 to 100 s^−1^ (logarithmic ramp).

The thixotropy test consisted of 2 equal cycles each comprising 3 steps. In the first step, the dynamic viscosity was acquired at a low and constant shear rate of 0.01 s^−1^ to simulate the at-rest viscosity. In the second step, the shear rate was set at a constant value of 80 s^−1^ to simulate the structural breakdown of the sample when subjected to an external force. The low shear rate used in the first step (0.01 s^−1^) was then reused in the third step to simulate the at-rest structural regeneration.

Then, the recovery (%) of the dynamic viscosity at different timepoints during the third step of the last cycle was calculated as an index of the structural restoration. The recovery (%) values were calculated as follows:

$$\mathrm{Recovery}\;\left(\%\right)=\frac{\eta_{i}}{\eta_{\left(1st\;step\right)}}\times 100$$

where η*_i_* is the dynamic viscosity value interpolated after “*i*” seconds of the third step (i.e., after 30 s, 60 s, 90 s, 120 s, and when the maximum recovery value was reached).

#### 2.1.4. Film Characterization

ODFs were evaluated by visual and organoleptic inspection. The average weight of ODFs was calculated as the average weight of 10 ODFs.

Analysis for determination of iron and folic acid was performed by Neotron (Italy).

The iron content was determined using inductively coupled plasma optical emission spectroscopy (ICP-OES). An aliquot of sample was weighed into microwave digester tubes and mineralization was carried out in the presence of concentrated HNO_3_ and HCl. The solution obtained was then brought up to volume quantitatively and filtered in test tubes and suitably diluted. The solutions obtained were analyzed with the ICP-OES technique. Folic acid content was determined by high-performance liquid chromatography. The sample was extracted with water at 0.1% (*w*/*v*) ascorbic acid and dithiothreitol at basic pH and acetonitrile in an ultrasonic bath. The final extract was brought to an acidic pH by adding concentrated H_3_PO_4_. The sample, suitably diluted and filtered, was analyzed using liquid chromatography with a diode array detector (HPLC-DAD, Agilent 1290, Santa Clara, CA, USA). The chromatographic column was an Ascentis Express C18 SB (10 cm × 3.0 mm; 2.7 µm) (Supelco, Bellefonte, PA, USA).

The disintegration test was performed according to the specifications of the orodispersible tablet reported in European Pharmacopoeia (Ph. Eur.) edition (2.9.1) in water at 37 °C using the Copley DTG100i disintegration tester (Copley Scientific, Nottingham, UK). To evaluate microbiological contamination, total aerobic microbial count, total yeast and molds count, and presence of *Escherichia coli*, *Staphylococcus aureus*, or *Pseudomonas aeruginosa* was determined.

#### 2.1.5. Stability Study

The stability of iron ODF was investigated at long, intermediate, and accelerated term conditions: 25° ± 2 °C and 60% ± 5% relative humidity (RH), 30° ± 2 °C and 65% ± 5% RH, and 40° ± 2 °C and 75% ± 5% RH, respectively. Each ODF was individually packed in a polyethylene terephthalate (PET)/foil extrusion laminate sachet and stored in climatic chambers in the primary packaging. At determined timepoints, ODFs were evaluated for aspect, average weight, iron content, folic acid content, disintegration, and microbiological presence.

### 2.2. Comparative Kinetic Pilot Study

#### 2.2.1. Food Supplements for the Kinetic Pilot Study

To evaluate iron absorption in the kinetic pilot study, Sucrosomial^®^ iron capsules enriched with 70 mg vitamin C (SiderAL^®^ FORTE, PharmaNutra Spa) were chosen as the reference product. Sucrosomial^®^ iron is ferric pyrophosphate protected by a phospholipid bilayer and a sucrester matrix (sucrosome) [33]. This technology allows iron to pass intact through the gastric environment and to be absorbed in the intestine, demonstrating strong tolerability and a high bioavailability in the body. The reference contained the same amount of elemental iron as iron ODF (test product; 30 mg iron from ferric pyrophosphate), and it contained vitamin C (which is not present in the test product) but no folic acid (which is present in the test product).

#### 2.2.2. Participants

Healthy female volunteers (n = 10) aged 18–55 years were enrolled in the study following the collection of written informed consent and confirmation of capacity to understand the nature and purpose of the study, including possible risks and side effects. The capability of the participant to collaborate with the investigator and meet the requirements of the study was also assessed.

Participants with the following criteria were excluded from the study: smoker; clinically significant abnormalities in the ECG evaluation; clinically significant abnormal laboratory values indicative of disease; known allergy or presumed hypersensitivity to the food supplement investigated (iron) and/or to the excipients of the 2 formulations; history of anaphylaxis from drugs, dietary supplements, or allergic reactions in general that could influence the outcome of the study in the opinion of the investigator; significant history of kidney, liver, gastrointestinal, cardiovascular, respiratory, skin, hematological, endocrine, or neurological diseases that may interfere with the purpose of the study; use of herbal remedies and dietary supplements in the 2 weeks prior to the start of the study; use of corticosteroids, thyroid hormones, antibiotics, or antiepileptics; alcohol abuse; any clinical condition that in the judgment of the investigator was deemed incompatible with study participation; and pregnant or breastfeeding women.

#### 2.2.3. Study Design

The study was designed as a pilot, crossover, open-label, single-center, comparative clinical trial. The study aimed to determine the iron blood concentration over time (up to 8 h) after single-dose administration of iron ODF (test product) versus a Sucrosomial^®^ iron capsule (reference product).

The study was approved by the local Ethics Committee (Comitato Etico della Fondazione Policlinico Universitario Campus Bio-Medico) and was performed according to good clinical practice guidelines and the Declaration of Helsinki. The trial was registered on ClinicalTrials.gov, number NCT05660200. The study was conducted at the Operative Research Unit of Clinical Laboratory, Fondazione Policlinico Universitario Campus Biomedico, Rome, Italy, from November to December 2022.

Ten healthy volunteers were enrolled in the study. After signing the informed consent and eligibility criteria verification, participants were assigned to the predefined treatment sequence (test product-reference product or reference product-test product) according to a randomization list generated by an electronic procedure, which defined the order of administration of the 2 supplements. One participant dropped out after the first blood sample collection due to abnormal laboratory values (falling within the exclusion criteria).

**Figure 1:**
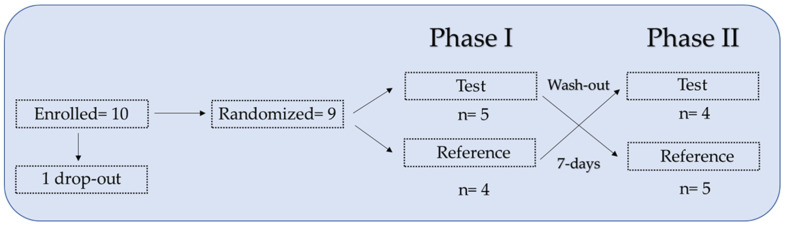
Schematic crossover study design with 10 healthy volunteers. Test, iron ODF. Reference, Sucrosomial^®^ iron capsule.

In the crossover setting, participants received both supplements with a 7-day washout between the 2 administrations. Phases I and II identify the timeframe corresponding to the 2 administrations, including the predefined visits and blood sampling. Both phases included 8 timepoints: 24 h before the first administration and, on the following day, at 0, 1, 2, 3, 4, 5, and 8 h. Phase II took place 7 days after Phase I (Figure 1). Peripheral blood samples were collected from each participant before (−24 h, 0 h) and after each administration (1 h, 2 h, 3 h, 4 h, 5 h, and 8 h).

In the week preceding the start of the trial and throughout the study period, including the 7-day washout period, the volunteers were instructed to follow nutritional recommendations for a balanced iron intake.

The administration of each supplement occurred on day 0 of each phase in the morning and on an empty stomach. The primary endpoint was the extent (area under the concentration–time curve from time 0 h to time 8 h, AUC_0–8_), the maximum concentration (C_max_), and the time corresponding to the maximum concentration (T_max_) of serum iron. The secondary endpoints were the AUC_0–8_, C_max_, and T_max_ of serum folic acid and blood parameters related to iron metabolism (ferritin, transferrin, transferrin receptor, hemoglobin, hematocrit).

During the study, the safety and tolerability of the 2 supplements were recorded based on the following evaluations: recording of adverse events, measurement of vital signs (blood pressure, heart rate, and temperature) at predefined timepoints (−24 h, 0 h, 4 h, and 8 h), and measurement of hepatic and renal function parameters (aspartate aminotransferase, alanine transaminase, gamma-glutamyl transferase, blood urea nitrogen, and creatinine).

#### 2.2.4. Sample Size and Statistical Analysis

The sample size (n = 10, with 1 drop-out) was decided arbitrarily considering the exploratory–descriptive nature of this pilot study. All data were described by quantitative statistics (mean, median, standard deviation, variance, and interquartile range). Data were analyzed with Stata 16 software (StataCorp LLC, Lakeway Drive, College Station, TX, USA).

## 3. Results and Discussion

### 3.1. Development and Characterization of Iron ODF

#### 3.1.1. Selection of Nutrients

Different salts as sources of iron were evaluated, namely, ferrous citrate, lactate and bisglycinate, and ferric pyrophosphate. These forms of iron were selected because they are permitted by European regulation 1170/2009, Annex II [35] for the use in food supplements. Table 1 lists the main characteristics of the iron salts considered. The iron salts are thin powders with a light color and acceptable taste. The percentage of elemental iron ranged from 19% to 25%, and consequently, the amount of salt necessary to have 30 mg of iron in the film was very high (120 mg to 150 mg).

Italian regulations (similar to other European regulations) establish 30 mg as the maximum dose of daily iron intake as supplementation, which corresponds to 214% of the nutrient reference value (NRV) [36].

Several ODF formulations were prepared using a percentage of iron salt on the dried mass of 30% weight per weight (*w*/*w*). Ferrous lactate was eliminated because of its taste (an unpleasant and persistent metallic taste appeared during disintegration in the oral cavity). The formulation containing ferrous bisglycinate became dark and almost black, and this aspect of the film could be unpleasant. ODFs prepared with ferrous citrate and ferric pyrophosphate were found to be the most pleasant. Ferric pyrophosphate was more palatable than citrate salt; moreover, the percentage of the iron in the salt was more favorable, enabling a lower amount of salt required for 30 mg of elemental iron (120 mg of ferric pyrophosphate versus 142 mg of ferric citrate). Considering these more favorable parameters, ferric pyrophosphate was selected as the iron source.

Iron and folic acid are often prescribed together, especially to pregnant women for whom the WHO recommends the daily consumption of both nutrients [37]. The NRV for folic acid is 200 µg/day, and the amount selected for this formulation was 400 µg/film corresponding to 200% of NRV. In compliance with Italian regulation, a 20% overdosage of folic acid was applied in the formulation. Table 2 reports the health claims of iron and folic acid by European regulations [38]. Elemental iron contributes to several physiological processes, such as the production of red blood cells and hemoglobin, oxygen transportation, and normal cognitive performance. In addition, folic acid contributes to homocysteine metabolism, the growth of maternal tissues during pregnancy, and to hematopoiesis. Together, iron and folic acid are also involved in cell division and contribute to the proper performance of the immune system and a reduction in tiredness and fatigue [39].

#### 3.1.2. Selection of Excipients and Formulation Development

In general, the main components of ODFs are, in addition to active substances, (i) the film-forming polymers, which can be natural or synthetic, (ii) plasticizers that improve the mechanical properties of the film, (iii) surfactants, and (iv) flavor and sweeteners to mask the taste of active substances [30]. Table 3 reports all excipients tested in the formulation development with their relative functions.

Iron ODF was based on IBSA FilmTec^TM^ technology and originated from lessons from the patents WO2005/039543 (“Self-supporting film for pharmaceutical and food use”) and WO2014/049548 (“ODFs having quick dissolution times for therapeutic and food use”) [40,41], where MDX as the only film-forming ingredient is used. MDXs are natural, safe, and cheap polymers widely used in pharmaceutical and food applications. They are water-soluble, film-forming biopolymers and, according to these patents, an amount of MDX ranging from 40% to 80% allows the formation of a rapidly dissolving, self-supporting, and edible film. This polymer provides advantages of palatability, physical properties, and stability. MDXs are water soluble, and no other organic solvents are necessary for film preparation. MDX was successfully previously used to design pharmaceutical oral film formulations containing sildenafil sitrate and vitamin D3 [25,42,43]. In vivo studies showed that such formulations were safe, efficacious, and bioequivalent to the conventional branded film-coated tablets [42]. Similar technology, but with the addition of a low amount of a second film former, was applied to a food supplement; an ODF containing 2000 IU of vitamin D3 was developed and manufactured on an industrial scale [25]. In iron ODF formulation, MDX was introduced at 34.5%.

To improve the mechanical properties of MDX film it is mandatory to add plasticizers. Plasticizers improve the flexibility and reduce the brittleness of ODFs, lowering the polymer glass transition temperature. Plasticizers also help overcome brittleness after the drying process. For iron ODF, sorbitol and glycerol were tested as plasticizers, and both gave suitable mechanical properties when added at 10–12%. Glycerol, however, dispersed the iron salt in the mixture better than sorbitol. Consequently, the ODF obtained using glycerol was smoother and the sensation of roughness during disintegration in the oral cavity was minimized.

Surfactants were added during the coating step to improve the spreadability of the water-based mass on a silicone/PET-release liner. Based on previous findings, [25] glyceryl monolinoleate and polysorbate 80 were chosen as surfactants. Glyceryl monolinoleate consists of mono-, di-, and triglycerides of mainly linoleic (C18:2) and oleic (C18:1) acids and is a water-insoluble surfactant with a hydrophilic-lipophilic balance (HLB) of 1. Polysorbate 80 is a non-ionic surfactant and viscous water-soluble yellow liquid with an HLB of 15. These surfactants were mixed to obtain an HLB of six (suitable for the coating step and to avoid foaming during mixing).

Patent WO 2014/049548 reports that it is possible to avoid hardening of the films based on MDXs and plasticizers by adding a homopolymer or copolymer of vinyl acetate to the composition [41,44]. According to this patent, copovidone (PVP-VA) was added as filler in the formula at 2%.

Adding flavors and sweeteners was considered to improve the taste of the iron ODF. Several flavors were tested but lemon flavor was most effective at masking the iron metallic taste. Sucralose, acesulfame K, and sodium cyclamate were combined according to the limits of the European Regulation on food additives [45] to create a favorable taste in association with natural lemon flavor. No color or opacifier was necessary because the high presence of iron made the film naturally opaque and yellow. A citrate buffer at a pH of 3.5 was added by combining citric acid and sodium citrate. Adding an acid can improve the bioavailability of iron [46].

Major drawbacks of ODFs, as described in the literature, are the limited amounts of active substance that can be loaded and the unpleasant taste of most active substances. In the iron ODF, it was possible to load a high amount of active substance (up to 35.3%) to reach the target dosage strength of the iron and the notoriously unpleasant taste of iron was well masked by adding lemon flavor and sweeteners.

#### 3.1.3. Manufacturing Process

**Figure 2:**
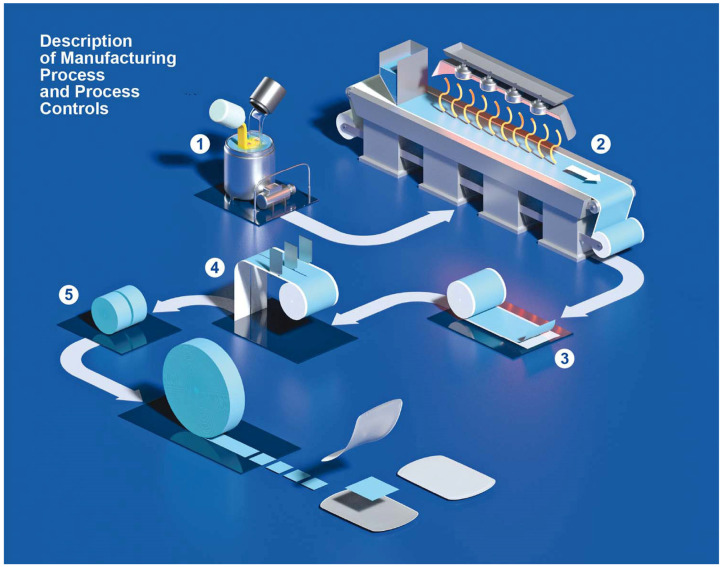
Description of the ODF industrial manufacturing process. The solvent casting method used to manufacture ODFs consisted of several steps. 1: A liquid mixture was prepared (temperature and stirring speed are the critical process parameters). 2: The mixture was spread and dried in a drying tunnel by controlling temperature, air circulation, humidity, and coating speed. 3: The dried and spread-out mixture was wound to form a big roll, known as a jumbo roll. 4: The jumbo roll was unwound to underwent two consecutive cutting phases where the size of the film strips was selected, and the final dosage was defined. 5: The films are punched, pouched, and sealed in suitable single-dose sachets. Reproduced with permission from Cupone et al. [30].

**Figure 3:**
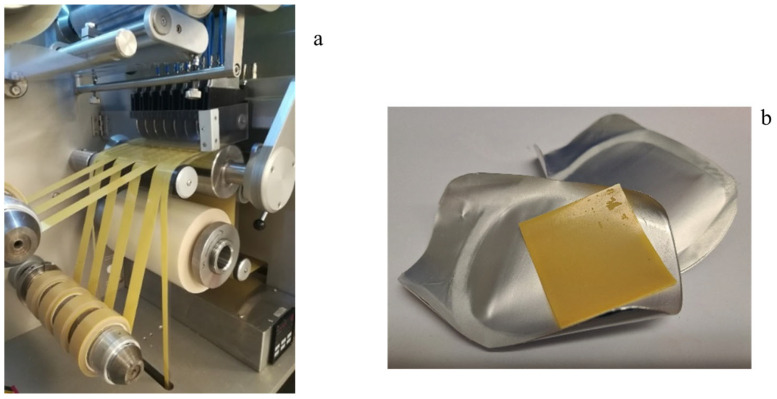
A spread and dried mass cut into reels (**a**), iron ODF (**b**).

Manufacturing of iron ODFs was performed according to an IBSA standard process developed and validated for the manufacturing of other ODFs already on the market, such as the pharmaceutical product sildenafil ODF [42] or 1000 IU and 2000 IU vitamin D3 films (as food supplements) [25]. The manufacturing process of iron ODF was scaled up from laboratory to industrial scale at the end of the pharmaceutical development study. Figure 2 shows the steps of the OFD manufacturing process in detail [30]. The mixture was prepared in a stainless-steel mixer with an anchor agitator and a high disk disperser. The mixture was pumped from the tank to the coating machine and spread using a knife on a siliconized support moving along the oven. At the end of the drying step, the laminate was cut into reels of 30 mm (Figure 3a). The reels were cut using a single rotary die blade with a cutting pitch of 30 mm that formed the final ODF, 30 × 30 mm (Figure 3b). The size of the ODF was defined by consecutive processes of slitting and formation. Each ODF was inserted between two packaging foils and sealed at two sealing stations.

During the scale-up of the manufacturing process, the following critical issues were identified:

- Introduction of a high amount of ferric pyrophosphate in the mixture during mass preparation.
- Viscosity of the mass during the coating process (see Section 3.1.4).
- Weight of the laminate obtained during coating and drying of the mass.

The first step in the preparation of the mixture is the dispersion of ferric pyrophosphate in the buffer solution containing glycerol and surfactant, using both the anchor mixer and a high disk disperser at a high-speed level. The large amount of the insoluble powder and the incorrect dispersion in the mixture can lead to a lack of homogeneity and an undesired crumbly aspect and sensation in the mouth. The amount of powder added is also responsible for the high viscosity of the mass. This step was carefully fine-tuned to allow for the homogeneous dispersion of the insoluble ferric pyrophosphate powder. An incorrect dispersion of ferric pyrophosphate can cause a non-homogeneous film and decrease its pleasantness because it emphasizes the sensation of powder in the mouth during the disintegration of the film in the oral cavity.

The weight of the dry laminate of iron ODF was settled at 377.8 g/m^2^ to obtain the required dosage. The weight of the film with ferric pyrophosphate was high when compared with other products on the market in the same dosage form, such as vitamin D3 ODF (167 g/m^2^) [25]. Considering that the percentage of water in the wet mixture was 40%, the weight of the wet mass coated on the liner was calculated as 561.0 g/m^2^. Mass flow rate, speed of the coating process, and temperature of the drying process were optimized to remove excess water but leave a suitable amount to plasticize the iron ODF.

#### 3.1.4. Evaluation of Rheological Properties of the ODF Wet Mixture

The viscosity of the wet mass is an important parameter that can affect the coating process. Wet mass is exposed to a wide range of shear rates and may have different issues and outcomes depending on its flow behavior. In fact, the rheological properties of the polymeric mixture affect the blending phase (especially the pumpability of the wet mass), drying rate, casting thickness, morphology, and content uniformity of the films [47,48].

Consequently, the influence of the formulation ingredients on the rheological properties of the wet mass must be studied. Typically, the following traits are desired:

- At rest, the wet mass should have a suitable viscosity to ensure physical stability (i.e., avoiding flocculation, creaming, or precipitation of the dispersed phase).
- When subjected to shear rates, such as during pumping, it has an acceptable dynamic viscosity to be processed without the need for high forces.

In addition, wet mass structural variations must allow for a reduction in viscosity during mixing which is reversible in an acceptable timeframe to ensure total restoration of initial conditions.

Previous studies on ODFs demonstrated that the suitable viscosity range is 2000–5000 mPa·s. For instance, the wet mass intended for the manufacturing of the vitamin D3 ODF food supplement was formulated to have a water content of 39.0% and a viscosity of approximately 3500 mPa·s, which was proven suitable for the industrial manufacturing process [25]. Mass-containing iron had a high solid-insoluble content due to the presence of iron pyrophosphate and appeared as a semisolid (i.e., a paste) that became thicker when not mixed.

**Figure 4:**
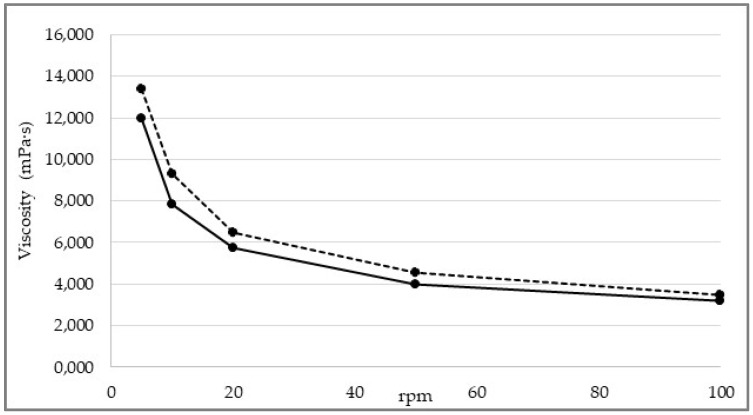
Viscosity of the ODF wet mixture as measured by changing test conditions. Dotted line: sample collected at the end of the coating step. Solid line: sample collected at the start of the coating step.

The viscosity of the mixture, measured with a Brookfield DV-E viscometer (Middleboro, MA, USA), ranged from 14,000 cP (LV4, 5 rpm) to 3000 mPa·s (LV4, 100 rpm) depending on test conditions (Figure 4). The rheological properties of the mixture were investigated in-depth. How the dynamic viscosity of the mixture varies as a function of the applied shear rate (γ̇) and whether and how its structure returns to the initial state when applied stress ceases were evaluated, enabling further considerations on the correlation between rheological properties of the mixture and machineability to be made.

**Figure 5:**
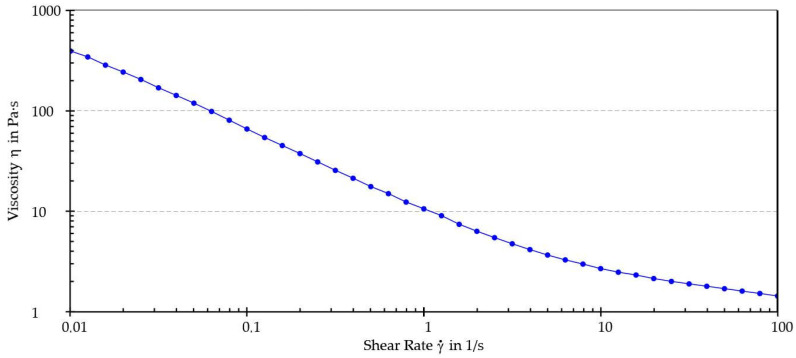
Viscosity curve.

Figure 5 illustrates the viscosity curve while Table 4 shows the dynamic viscosity values interpolated from the viscosity curve at shear rates of 0.01, 0.1, 1, 10, and 100 s^−1^.

**Figure 6:**
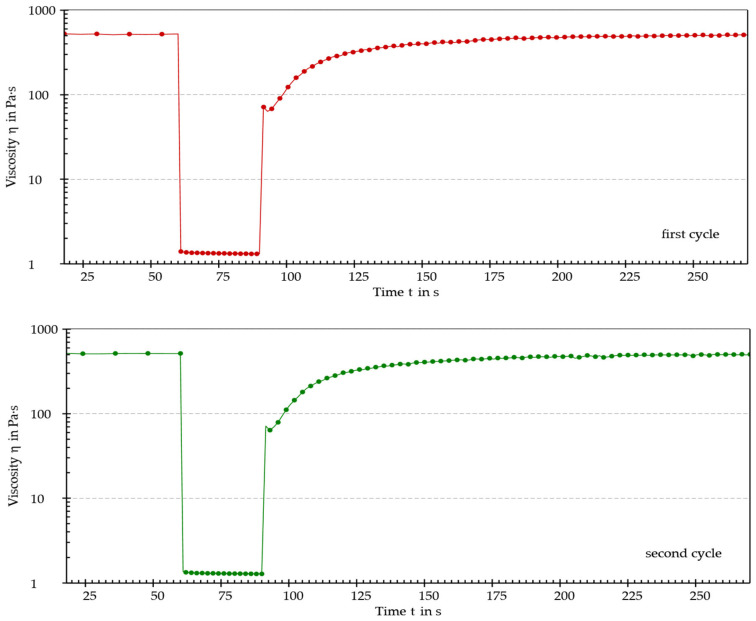
The two cycles of the thixotropy test.

**Figure 7:**
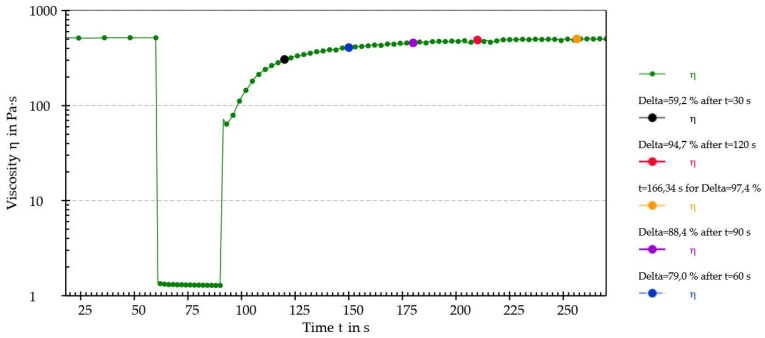
The last cycle of the thixotropy test with interpolated points highlighted using blue dots.

As shown by the results, the sample exhibited shear-thinning behavior. Furthermore, an increase in shear rate was associated with a decrease in dynamic viscosity. This behavior, typical of pseudoplastic fluids, generally occurs because of orientation and, eventually, disentanglement (i.e., as in the case of macromolecules) of the dispersed phase in the direction of the applied stress. A thixotropy test was performed to determine whether the sample could recover its original structure after being subjected to external stress. The thixotropy test diagrams are shown in Figure 6 and Figure 7 and recovery (%) values are reported in Table 5.

The sample achieved almost total regeneration of the initial structure after 120 s, thus displaying thixotropic behavior. Considering the obtained results, it was possible to establish that the analyzed mixture of iron ODF was a pseudoplastic and thixotropic fluid. Since these properties are generally targeted when considering machinability, it was concluded that the intermediate wet mass of iron ODF had optimal flow properties for the manufacturing process.

#### 3.1.5. Characterization of Iron Orodispersible Films

**Figure 8:**
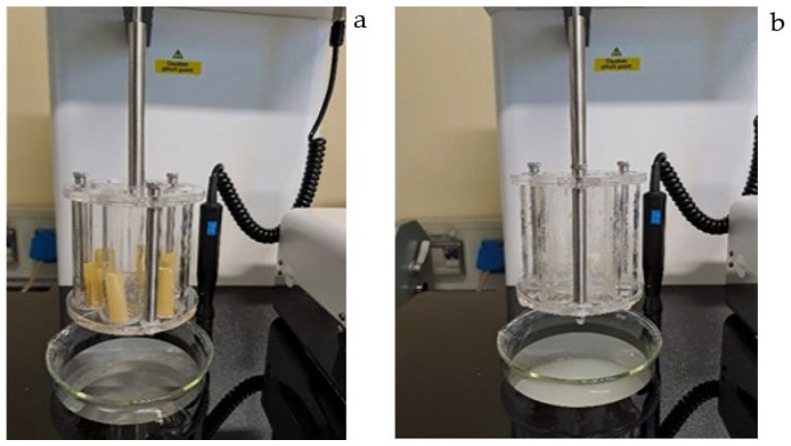
Disintegration apparatus at the start of disintegration test (**a**) and at the end (**b**).

Critical quality attributes (CQAs) of iron ODFs were identified and investigated (Table 6). The films obtained were thin, flexible, homogeneous, yellow, and characterized by a pleasant odor and taste of lemon fruit. Films were homogeneous for iron and folic acid content and disintegrated in less than three minutes. Figure 8 shows disintegration apparatus at the start (a) and at the end (b) of the disintegration test of iron ODFs. At the end of the test, films appeared completely disintegrated. Specifications for microbiological contamination were specific to ODFs. No microbial contamination was observed. Iron ODFs are gluten and lactose free, making them suitable for people with food intolerances.

The maximum dimension of film was set to 3 × 3 cm to ensure ease of handling and consumption.

#### 3.1.6. Stability Results for Iron Orodispersible Films

A stability study that lasted at least six months was carried out according to ICH guideline Q1 A (R2) “Stability Testing of new Drug Substances and Products”, which is the current effective version [49]. An accelerated stability study at 40 °C and 70% RH and intermediate conditions (30 °C and 65% RH) was conducted to stress the formulation, and a long-term stability study at 25 °C and 60% RH was started. All the CQAs of iron ODF were investigated during the stability study and a 24-month minimum shelf-life was required. All test results were not markedly different from the starting point value; films preserved their physical properties and remained thin, flexible, homogeneous, and yellow, and had a characteristic odor of lemon fruit and disintegrated rapidly. The moisture content of ODF did not change with temperature and humidity, showing that the material selected for the primary packaging was suitable for storage of the product.

**Figure 9:**
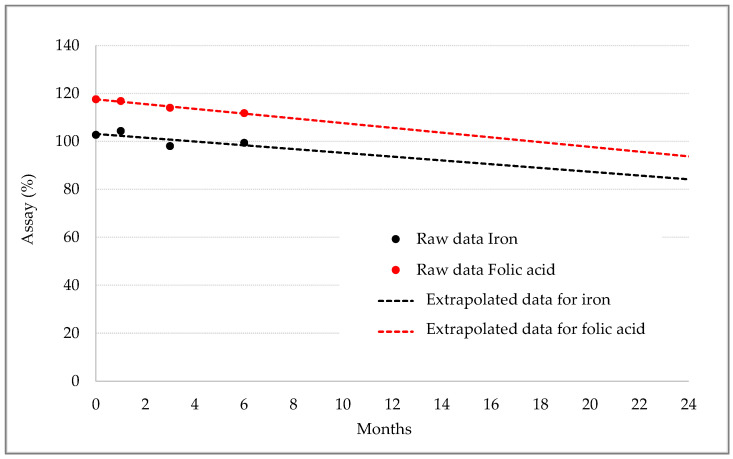
Stability trend for iron ODF.

Table 7 shows stability data for iron and folic acid contents at different timepoints. Iron content fluctuated around 100% without marked differences in different storage conditions, and folic acid content at six months decreased proportionally with the temperature but was still within the required specifications (Table 7). The extrapolation of the data, shown in Figure 9, indicated that the content of both nutrients would still be within the required specifications for up to 24 months.

### 3.2. Comparative Kinetic Pilot Study

#### 3.2.1. Participants

A total of 10 healthy participants were enrolled in this pilot study and assigned to a treatment sequence test product-reference product or reference product-test product according to a predefined randomization list. One participant dropped out of the study after the first blood sample was collected due to abnormal laboratory results (deemed as compatible with the exclusion criteria). Of the nine remaining volunteers, five followed the test product-reference product sequence and four followed the reference product-test product sequence.

The average age of the enrolled participants was 22.8 ± 2.2 years. Median (interquartile range; IQR) body measurements were as follows: weight, 56 kg (55–57); height, 165 cm (164–168); and body mass index, 20 (IQR 20–21). Vital signs of participants (blood pressure, heart rate and temperature) were within the normal range before and after administration of each iron supplement. No major protocol deviations were reported.

#### 3.2.2. Primary Endpoint Findings

**Figure 10:**
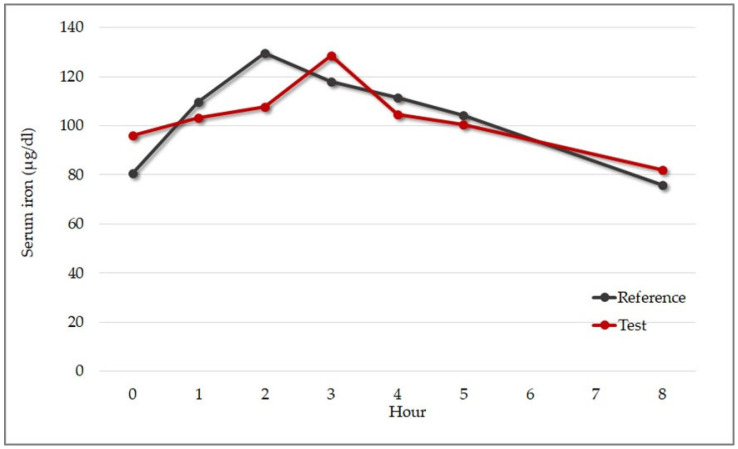
Mean change in serum iron concentration from baseline over 8 h after administration of 30 mg of test or reference product (n = 9). Test product: red line. Reference product: gray line.

The concentration of serum iron was measured at multiple timepoints to evaluate the rate and extent of iron absorption after consumption of a single oral dose of test versus reference product: −24 h, 0 h (pre-administration), and at 1 h, 2 h, 3 h, 4 h, 5 h, and 8 h post-administration). Maximum serum concentration (C_max_), time to achieve C_max_ (T_max_), and area under the concentration–time curve (AUC_0–8_) were assessed. Figure 10 shows the mean serum iron concentration over time (from 0 h pre-administration to 8 h post-administration) before and after consumption of the test versus the reference product.

The mean AUC_0–8_ was 824.37 µg × h/dL for the test product and 836.97 µg × h/dL for the reference product, and C_max_ was 136.33 µg/dL for the test product and 131.22 µg/dL for the reference product. The median time of maximum concentration (T_max_) was two hours for the reference product and three hours for the test sample (Table 8)**.** There was no obvious explanation for the difference in T_max_ between the products; however, concentration–time curves almost overlapped at four hours post-supplementation, and they both returned to baseline levels at eight hours.

The bioequivalence of both products was evaluated with the Anderson and Hauck’s test and Schuirmann’s two one-sided tests. While the Anderson and Hauck’s test suggested a trend towards bioequivalence (*p* < 0.05), Schuirmann’s two one-sided tests (*p* = 0.10) supported the hypothesis of non-equivalence. Equivalence tests require larger sample sizes to ensure adequate power, so the results were merely descriptive. Nonetheless, the serum iron concentration–time curves showed a similar trend that needs confirmation with larger trials.

#### 3.2.3. Secondary Endpoint Findings

The effect of both supplements on the biomarkers indicative of iron metabolism was then evaluated through descriptive analysis. Specifically, hemoglobin, hematocrit, ferritin, transferrin, and transferrin receptor in whole blood were assessed at defined timepoints pre- and post-supplementation and no relevant differences were observed between the two supplements.

**Figure 11:**
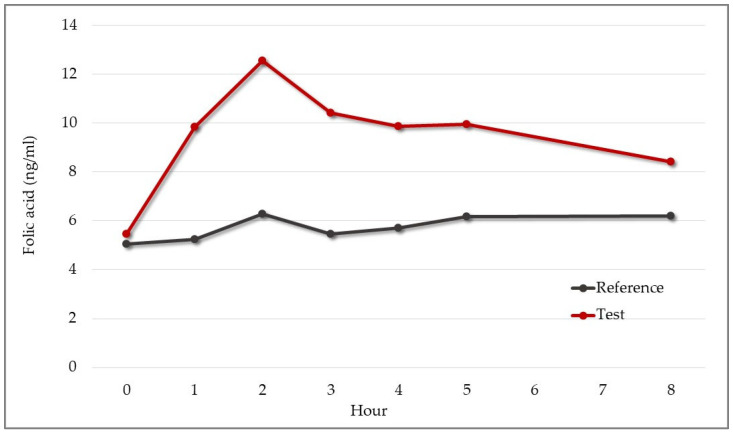
Mean change in serum folic acid from baseline over eight hours after administration of 30 mg test or reference product (n = 9). Test product: red line. Reference product: gray line.

Folic acid was contained only in the test product, and for this parameter, the reference product acted as a negative control. The rate and extent of folic acid absorption are shown in Figure 11. As expected, the mean concentration of folic acid in blood for participants who received the reference product showed minimal variations that were not relevant (Figure 11).

Regarding the concentration–time curve for the test product, the mean value of AUC_0–8_ was 79.8 ng × h/mL, C_max_ was 13.64 ng/mL, and the median T_max_ was two hours (Table 9).

The results provided the first and preliminary evidence regarding the absorption kinetic profile of folic acid in the test product.

#### 3.2.4. Safety Endpoint Findings

No alteration in vital signs and laboratory values (indicative of hepato-renal function) were reported. Likewise, no adverse events occurred during the study. The administration of both supplements was safe and well tolerated.

## 4. Conclusions

Ferric pyrophosphate combined with MDX as a film-forming polymer and glycerin as a plasticizer resulted in an ODF that disintegrated rapidly in the oral cavity without water, was easy to handle, and had good organoleptic properties. The manufacturing process was successfully transferred from the laboratory scale to the industrial scale, and the feasibility of industrial manufacturing of ODF was demonstrated. Based on current data, iron ODF can be considered stable for up to 24 months.

A major technological challenge was to load a large amount of iron and folic acid into the ODF formulation; nonetheless, we managed to reach the target of 30 mg of elemental iron corresponding to 120 mg of salt, representing 35% of the ODF composition, and quality target product profiles were satisfied.

Data from the bioavailability study with a crossover design showed a similar kinetic profile for iron ODF versus Sucrosomial^®^ iron capsules regarding iron absorption. AUC_0–8_ and C_max_ of serum iron were comparable and no relevant differences in the kinetic profile of all other parameters indicative of iron metabolism were observed. Both supplements had a good tolerability profile since there were no alterations in vital signs or hepato-renal function and no onset of adverse events during the study.

The results demonstrated that the iron contained in iron ODF is absorbed and its serum concentration peaks three hours after film consumption. Folic acid is also absorbed, showing a peak in serum concentration after two hours. The kinetic profile for folic acid was examined only regarding iron ODF as this ingredient was not contained in the reference product.

These data represent the first proof of iron and folic acid absorption concerning the newly developed ODF. The availability of these data is highly informative for the development of larger trials to evaluate the clinical efficacy and tolerability of the new food supplement.

Iron ODF represents a valid alternative to conventional dosage forms and may be preferred by individuals who need daily iron supplementation because of its convenience and ease of consumption.

## Figures and Tables

**Table 1:** Characteristics of iron salts.

Iron Salt	Flavor	Color	Molecular Weight	% of Iron	mg of Salt Equivalent to 30 mg of Iron
Ferrous bisglycinate C_4_H_8_FeN_2_O_4_	Slightly metallic, sweetish	Greenish-gray	203.96	19–23% Fe(II)	142 mg
Ferrous Citrate C_6_H_6_FeO_7_	Slightly metallic	Gray-green	245.95	21% Fe(II)	142 mg
Ferrous Lactate C_6_H_10_FeO_6_	Mild sweet, metallic	Greenish-white	270.04	20% Fe(II)	150 mg
Ferric pyrophosphate Fe_4_O_21_P_6_	Neutral, slightly sour, slightly astringent	Yellowish	745.21	25% Fe(III)	120 mg

**Table 2:** Health claims concerning iron and folic acid [38].

Nutrient	Claim
IRON	Contributes to normal cognitive function
	Contributes to normal energy-yielding metabolism
	Normal formation of red blood cells and hemoglobin
	Contributes to normal oxygen transport in the body
FOLIC ACID	Contributes to normal psychological function
	Contributes to normal homocysteine metabolism
	Contributes to normal blood formation
	Contributes to normal amino acid synthesis
	Contributes to maternal tissue growth during pregnancy
IRON AND FOLIC ACID	Contributes to the normal function of the immune system
	Contributes to a reduction in tiredness and fatigue
	Has a role in the process of cell division

**Table 3:** Components tested during formulation development.

Function	Component
Nutrient	Ferric pyrophosphate, ferrous bisglycinate, ferrous citrate, ferrous lactate, folic acid
Film forming	Maltodextrin DE, 6
Plasticizer	Glycerol, sorbitol
Surfactant	Glyceryl monolinoleate, polysorbate 80
Flavor	Natural lemon, chocolate, caramel, and raspberry
Sweeteners	Acesulfame K, sodium cyclamate, sucralose
Filler	Copovidone
Other	Citric acid, sodium citrate

**Table 4:** Dynamic viscosity values at different shear rates.

η [mPa s] at γ̇ of:				
0.01 s^−1^	0.1 s^−1^	1 s^−1^	10 s^−1^	100 s^−1^
393,005	65,937	10,602	2697	1440

**Table 5:** The recovery values at different timepoints in the third step (last cycle) of the thixotropy test.

Recovery (%) Values after:				
30 s	60 s	90 s	120 s	166 s
59.2	79.0	88.4	94.7	97.4

**Table 6:** Critical quality attributes for iron ODF.

CQA	Target
Appearance	Flexible, thin film, square, yellow, homogeneous, characteristic flavor of lemon
Size	3 × 3 cm
Average weight	340.0 mg
Iron assay	100% of label claimed
Folic acid assay	100% of label claimed
Disintegration time	<3 min
Microbiological contamination	Total yeast and mold count ≤ 10^2^ CFU/film Total aerobic microbial count ≤ 10 CFU/film *E. coli*, *S. Aureus*, *P. aeruginosa*: Absent
Gluten and lactose	Free

**Table 7:** Stability data of iron and folic acid content.

Test Product	Storage Condition	T = 0	1 Month	3 Months	6 Months
Iron assay results	25 °C/60% RH	102.7%	104.3%	98.0%	99.3%
	30 °C/65% RH		104.7%	94.0%	106.3%
	40 °C/70% RH		105.0%	100.0%	100.0%
Folic acid assay results	25 °C/60% RH	117.5% *	116.8%	114.0%	111.8%
	30 °C/65% RH		117.0%	112.3%	107.0%
	40 °C/70% RH		110.5%	105.3%	98.5%

* In compliance with Italian regulation, a 20% overdosage of folic acid was applied in the formulation.

**Table 8:** Kinetics of serum iron.

Measure		Test Product			Reference Product		
		Mean	Median	Variance	Mean	Median	Variance
AUC_0–8_	Area under the concentration–time curve (µg × h/dL)	824.37	676.95	80,406.8	836.97	824.08	75,572.3
AUC_exp_	AUC from 0 to ∞ using an exponential extension (µg × h/dL)	1974.69	1732.82	916,623	1851.49	1151.61	1,019,033
C_max_	Maximum concentration (µg/dL)	136.33	116	3512.25	131.22	115	1863.19
T_max_	Time of maximum concentration (hour)	3.22	3	4.94	2.11	2	1.36
T_last_	Time at last concentration (hour)	8	8	0	8	8	0

**Table 9:** Kinetics of serum folic acid. Area under the concentration–time curve (AUC), AUC from zero to ∞ using an exponential extension (AUC_exp_), maximum concentration (C_max_), time of maximum concentration (T_max_), and time at last concentration (T_last_). Results refer to the test product only.

Measure		Mean	Median	Variance
AUC_0–8_ (ng × h/mL)	Area under the concentration–time curve	79.8	76.14	146.57
AUC_exp_ (ng × h/mL)	AUC from 0 to ∞ using an exponential extension	232	241.82	2377.09
C_max_ (ng/mL)	Maximum concentration	13.64	13.8	3.44
T_max_ (hour)	Time of maximum concentration	2.33	2	1.75
T_last_ (hour)	Time at last concentration	8	8	0

## Data Availability

Data that underlie the results reported in this article are available from the corresponding authors on reasonable request.
